# Image statistics determine the integration of visual cues to motion-in-depth

**DOI:** 10.1038/s41598-022-12051-5

**Published:** 2022-05-13

**Authors:** Ross Goutcher, Lauren Murray, Brooke Benz

**Affiliations:** 1 Psychology, Faculty of Natural Sciences, University of Stirling, Stirling, FK9 4LA UK; 2 Psychology, University of Edinburgh, Edinburgh, EH8 9JZ UK

**Keywords:** Sensory processing, Motion detection, Human behaviour

## Abstract

Motion-in-depth perception is critical in enabling animals to avoid hazards and respond to potential threats. For humans, important visual cues for motion-in-depth include *changing disparity* (CD) and *changing image size* (CS). The interpretation and integration of these cues depends upon multiple scene parameters, such as distance moved, object size and viewing distance, posing a significant computational challenge. We show that motion-in-depth cue integration depends upon sensitivity to the joint probabilities of the scene parameters determining these signals, and on the probability of CD and CS signals co-occurring. Models that took these factors into account predicted human performance in speed-in-depth and cue conflict discrimination tasks, where standard linear integration models could not. These results suggest that cue integration is affected by both the uncertainty of sensory signals and the mapping of those signals to real-world properties. Evidence of a role for such mappings demonstrates the importance of scene and image statistics to the processes underpinning cue integration and the perception of motion-in-depth.

## Introduction

The perception of motion-in-depth is critically important for survival: determining whether an object is approaching or receding provides a fundamental means of assessing potential threats or avoiding hazards^1–4^. In humans, a number of visual^5–9^ and non-visual^10,11^ cues support motion-in-depth perception, including changes in binocular disparity, measured as either differences in relative disparity over time, or as interocular velocity differences^5,12–17^ and changes in retinal image size^18,19^. The combination of these changing disparity (CD) and changing size (CS) cues has been shown to improve judgements of motion-in-depth in a range of tasks, including the estimation of collision time^20,21^, and motion direction^9,22,23^. The processes underpinning the integration of these cues are uncertain, however, with early findings suggesting a simple averaging of CS and CD cues^8^. Contrary to this, more recent results have shown that large conflicts, where CS and CD measures arise from very different motions, result in a dissociation between cues^5,24^. Such dissociation is consistent with the robust cue integration found in other areas of depth perception^25,26^.

**Figure 1:**
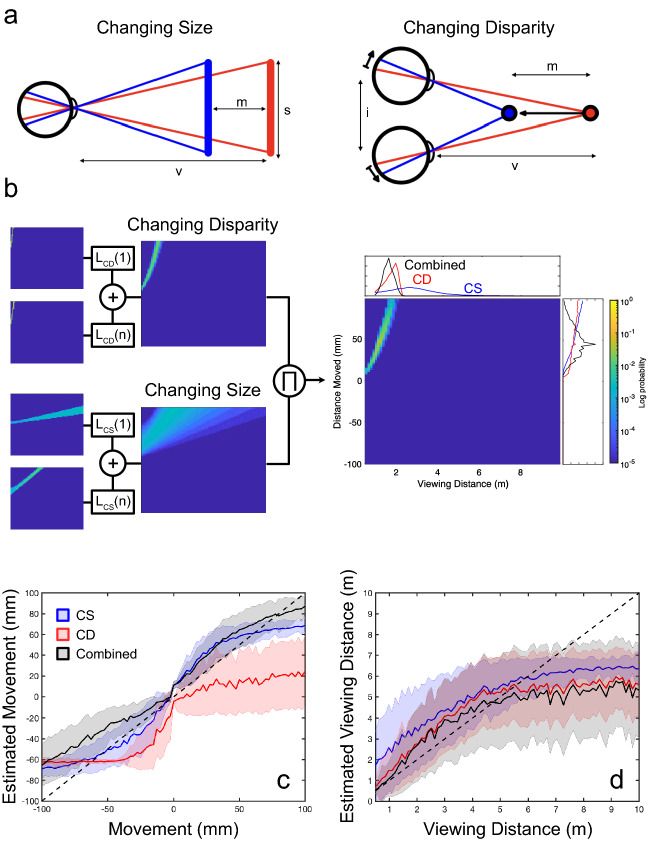
Motion-in-depth cues depend upon multiple scene parameters. (**a**) Motion-in-depth may be signalled by changing image size (CS), which depends on the viewing distance v, object size s and distance moved m, and on changing disparity (CD), which depends upon m, v, and the inter-ocular separation i. (**b**) Given these dependencies, Monte Carlo simulations of linear motion-in-depth reveal a probabilistic relationship between CS, CD, and the likelihood of joint values of m and v. These probabilistic relationships were used to model speed-in-depth perception. Scene parameter distributions for each cue were taken as the weighted sum of posterior distributions given uncertain CD and CS signals. Combined signals were taken as the normalised product of these weighted sum distributions, with scene parameter estimates given by the marginal means (see “Methods” for full details). (**c**,**d**) Resulting scene parameter estimates for distance moved and viewing distance, respectively, plotted against the true values. Blue, red and black lines show CS, CD and combined estimates, respectively. Shaded areas show standard deviations. These figures show that our model can successfully estimate values of m and v within a near-space range.

Measuring conflict between CD and CS is computationally challenging, however, since the interpretation of these cues is ambiguous: both cues depend on multiple scene parameters, including viewing distance, physical object size and distance moved (Fig. 1a). Thus, even in the absence of measurement noise, there exists no one-to-one mapping between CD and CS values and the scene parameters on which they depend. Signals for CS also depend upon the assumption that changing angular size is not due to changes in the physical size of an object^27–29^. Similarly, since CD depends on relative disparities^5,12,13^ (i.e., the disparity between multiple image points, independent of the viewing distance) it depends upon the assumption that only a single object in the scene is in motion. Together, these ambiguities mean that, during integration, the visual system is faced with the problem of determining if and how assumptions of object constancy (CS) and/or stationarity (CD) have been violated, while still facing more general problems of recovering speed and location in depth.

In this paper, we use Monte Carlo simulations of motion-in-depth to define posterior probability distributions for viewing distance and distance moved, given CD and CS values. We show that these distributions can be used to recover relevant scene parameters, with an associated model of speed-in-depth discrimination accounting for human psychophysical performance, including surprising deviations from the predictions of standard models of weighted linear cue integration^30,31^. We further show that statistical relationships between CD and CS signals account for the ability to determine when these cues are in conflict. Together, these results show that the statistics of image formation for motion-in-depth play a critical role in determining when and how CD and CS cues are integrated. Cue integration processes thus depend not only on the uncertainty of sensory signals themselves, but on the brain’s capacity to account for the uncertainty with which these signals map onto behaviourally relevant scene parameters.

## Results

To understand the mapping between scene parameters and CD and CS signals, we simulated one million linear motions for square, fronto-parallel surfaces moving towards or away from an observer, directly along the line of sight. Simulations were performed for a range of viewing distances, surface sizes and distances moved (see “Methods” for full details). These simulations were used to define posterior probability distributions p(v,m|CD) and p(v,m|CS) for combinations of viewing distance *v* and distance moved *m,* for a given CD or CS value. Note that, although CD calculations were based on disparity differences over time, this measure could be substituted for one based on interocular velocity difference without impacting the results. The mapping between world and image properties depends on the imaging process, not on any mechanism for encoding motion-in-depth. For the resulting CD and CS distributions, the range of probable viewing distances or distances moved decreased with increasing CD or CS (Fig. 1b).

These posterior distributions were used to model the estimation of motion-in-depth from integrated CD and CS signals for a new set of 10,000 simulations. Posterior distributions for a given pair of CD and CS values were multiplied together and renormalised to create combined cue posteriors. Final parameter estimates were taken as the marginal means for each parameter. Results from these simulations (Fig. 1c,d) show that the combination of CD and CS distributions provides reliable estimates of both the distance moved and the viewing distance, within a reasonable range. Measured r^2^ values ranged from 0.66 and 0.89 for CD and CS cues, respectively, to 0.90 for the combined cue model (p < 0.0005 in each case). Viewing distance was also reliably estimated, although only until around 6 m, suggesting that the value of CD and CS for motion-in-depth may be limited to comparatively near space.

To understand the relevance of this model for human vision, we conducted two experiments. The first examined cue integration for the discrimination of speed-in-depth, while the second focussed on the ability to detect CD and CS cue conflicts.

### Mapping of motion-in-depth cues affects speed-in-depth discrimination

Although our simulations demonstrated that a posterior distribution model could estimate motion-in-depth, this does not mean that human observers have access to such probabilistic representations. To determine whether human perception is consistent with our model, we conducted a speed-in-depth discrimination task, where CS reliability was manipulated by parametrically varying the size of the simulated surfaces presented to observers.

Participants were presented with a series of trials using a two-interval, perturbation analysis^30,31^ paradigm. Each interval contained a square, fronto-parallel surface oscillating forwards and backwards in depth along a triangular waveform. Each stimulus was textured with a random luminance checkerboard pattern, ensuring the availability of local edge structures and providing reliable signals for both CD and CS. In one interval, CD and CS were put into conflict by varying the amplitude of these oscillations for each cue. The other interval contained a conflict free, combined CD and CS stimulus, with participants asked to judge which of the two intervals contained the faster motion-in-depth. By parametrically varying the speed of the consistent cue interval, we measured bias and sensitivity (given by μ and σ parameters of a fitted cumulative Gaussian, where μ is the point of subjective equality, PSE) for speed-in-depth discrimination for surface sizes of 10 × 10, 40 × 40 and 60 × 60 mm.

**Figure 2:**
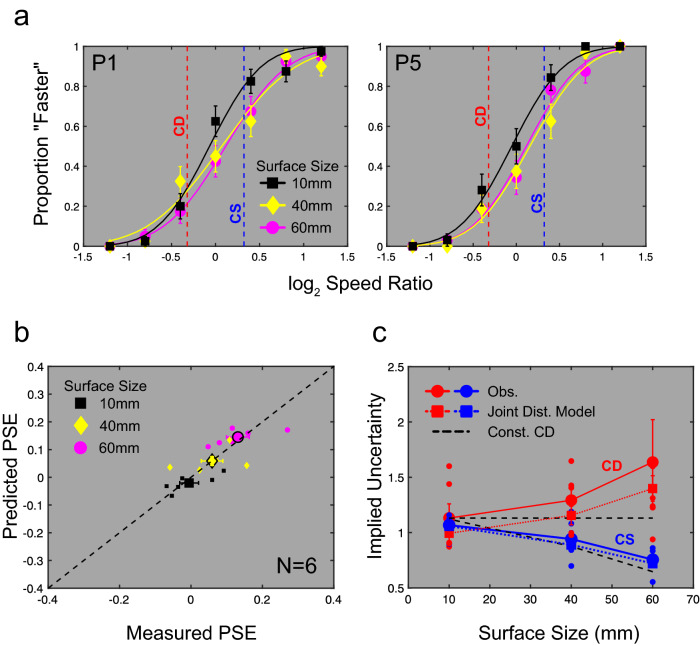
Human speed-in-depth discrimination is accounted for by the joint distribution model. (**a**) Psychometric functions for 2 example observers show shifts in PSE towards the CS cue with increasing surface size. (**b**) Shifts are well-predicted by our joint distribution model, across all participants (smaller symbols show individual participants, larger symbols show mean performance). (**c**) Implied cue uncertainties as a function of object size for CD (red) and CS (blue) cues. Continuous lines with large circles show implied uncertainties averaged across observers. Error bars show the standard error on the mean. Small circles show individual implied uncertainties. Coloured dotted lines with large squares show the implied uncertainties predicted by the joint distribution model. Black dashed lines show the expected change in implied CS uncertainty when CD uncertainty is held constant (see “Methods” for details). Our model predicted observed changes in implied cue uncertainty that would otherwise require ad hoc changes to standard cue integration models.

The results of this perturbation analysis experiment are shown in Fig. 2a as fitted psychometric curves, normalised for direction of cue conflict. These curves show a significant shift in PSE towards the CS cue with increasing surface size (*F*_2,10_ = 8.256, *p* = 0.008, ${\eta }_{partial}^{2}=0.623$ on a Repeated Measures ANOVA).

To apply our modelling approach to human speed-in-depth discrimination, we introduced uncertainty to initial CD and CS measurements. Uncertainty was introduced to estimates of disparity and image size, sampled every 5 frames along each cue’s triangular oscillation in depth, by considering these estimates as unbiased Gaussian distributions with standard deviations *n*_*disparity*_ and *n*_*size*_, respectively. CD distributions were defined as the difference between these uncertain disparity measurements, while CS distributions were defined as the ratio of uncertain size measurements^32,33^.

The resulting distributions of CD and CS values were used to produce posterior distributions for the normalised sum of weighted distributions for each cue (Fig. 1b). Speed-in-depth estimates were found by taking the marginal mean of combined cue distributions with respect to distance moved (see “Methods” for full details).

We fit this model to human task performance, with *n*_*disparity*_ and *n*_*size*_ as free parameters. Model performance matched the effects of increasing surface size on the location of the PSE (*r*^2^ = 0.61, *p* = 0.0001) (Fig. 2b). Although, at an individual level, these fits do not fully account for the range of observed PSE shifts, they do provide an excellent fit to the average shifts across observers. Best-fitting values of *n*_*disparity*_ and *n*_*size*_ averaged 0.35 and 3 arcmin, respectively, across participants. Note that the value of the PSE shifts with increasing surface size despite the fitted *n*_*size*_ value remaining constant. This is because increased surface sizes produce larger CS values, reducing the contribution of *n*_*size*_ related image-to-image size uncertainties.

In addition to fitting observed PSE shifts, model performance also matched psychometric function slopes for each observer. Typically, studies of sensory integration have predicted the slope of combined cue functions based on an optimal weighted averaging of individual cues^30^, where these thresholds are interpreted, in a parameter free fashion, as the uncertainties associated with independent sensory signals. Such an approach is particularly problematic for studies of motion-in-depth, where single cue stimuli necessarily entail large cue conflicts that could impact upon integration processes. To address this issue, we adopted an alternative approach. Rather than use the measured uncertainty of individual cues to predict combined cue thresholds, we inferred the individual cue uncertainties consistent with observed thresholds and PSE locations for combined cue stimuli, under the assumption that these were consistent with standard linear integration approaches^30,31^. We term this the *implied cue uncertainty.* This is defined by the relationship between cue weights and individual cue thresholds, where the ratio of PSE derived cue weights ${w}_{CD}/{w}_{CS}$ equals the ratio of observed variances ${\sigma }_{CS}^{2}/{\sigma }_{CD}^{2}$, and where *w* is the cue weight, and σ is the standard deviation of the psychometric function^26^. Using these relationships, we can express the implied cue uncertainties for ${\sigma }_{CS}^{2}$ and ${\sigma }_{CD}^{2}$ using only terms directly measurable from our psychophysical data (see “Methods” for full details and derivation).

Implied cue uncertainties were calculated for CS and CD cues at each surface size for human observers and for our joint distribution model (Fig. 2c). Surprisingly, these results show that an increase in surface size not only decreased the implied uncertainty of the CS cue, but also increased the implied CD uncertainty. While this is contrary to the behaviour of standard models of linear cue integration, where CD uncertainty should remain constant, this behaviour is well matched by the joint distribution model (*r*^*2*^ = 0.75, *p* < 0.0005). As a further comparison, we calculated the change in CS uncertainty required to account for our findings, if the CD uncertainty were constant, as expected from our manipulation of surface size. Assuming a constant CD uncertainty at the level found for the 10 mm surface, implied CS uncertainty is underestimated by 0.11 log_2_ units for the 10 mm surface compared to human performance. For the 60 mm surface, this changes to an overestimation of 0.05 log_2_ units. This measure provided a clear poor match to human behaviour (r^2^ = 0.58, p = 0.08).

Note, the observed changes in implied CD uncertainty do not result from any actual change in the uncertainty of the CD cue. Instead, changes in implied uncertainty occur due to a combination of the CS signal uncertainty *n*_*size*_*,* which reduces with increasing surface size, and the uncertainty with which these signals map onto real world motions. Thus, while increases in surface size decrease the uncertainty of CS signals, this does not translate into an equivalent reduction of uncertainty when one considers the mapping of these CS signals onto motions in the world. Given observed changes in PSE locations, combined cue thresholds do not improve to the extent expected by standard models of linear integration. These non-standard effects may arise when cues take the form of skewed distributions^34^. Here, mapping uncertain CS signals onto our joint distributions for distance moved and viewing distance resulted in positively skewed distributions, where slower speeds are more likely to occur. This effect is reduced at larger surface sizes, where the change in image size is both larger and more reliable.

### Cue conflict discrimination determined by cue co-occurrence

**Figure 3:**
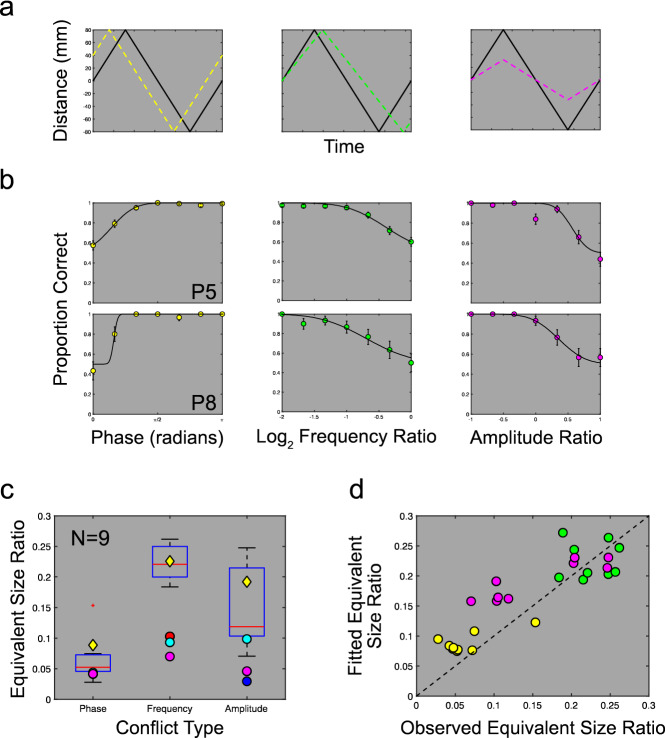
Co-occurrence statistics explain human cue conflict discrimination. (**a**) Illustration of the three conflict types applied in Experiment 2. Colour coding for phase (yellow), frequency (green) and amplitude (magenta) conflicts are preserved in b and d. Dashed coloured lines show CS, black lines show CD. (**b**) Psychometric functions for two example participants for each conflict type. (**c**) To compare conflict types, we found the equivalent physical size change that would have produced the observed threshold-level cue conflict. This was summarised as the ratio of CD to CS surface sizes, taking the absolute maximum on a log_2_ scale. Boxplots show the mean and range of equivalent size ratios found across participants. Yellow diamonds show the results of this analysis for the cue co-occurrence model, which provided a good fit to human observers. Results are also shown for joint distribution model variants that measured the standard deviation and maximum differences (blue; red), or ratios (cyan; magenta), in estimated speed-in-depth between cues (variants based on physical size estimates performed at chance level and are therefore not plotted). (**d**) Equivalent size ratios for each conflict type, for each participant, plotted against fits for the cue co-occurrence model.

While our joint distribution model successfully accounted for speed-in-depth discrimination, such performance assumes that the integration of CS and CD cues is guaranteed to occur. Limits on cue integration have been observed in tasks related to the perception of 3D shape^25,26^ and may be particularly important for motion-in-depth, where large conflicts between CS and CD cues can arise with changes in physical object size or with the motion of multiple objects. The uncertain mapping between physical motion in the world and resulting CS and CD signals further complicates this integration process. We measured limits on cue integration by asking participants to perform a conflict discrimination task, where cue conflict leads to the perception that the stimulus is changing in physical size. As in Experiment 1, participants were presented with two intervals, each containing triangular wave motion-in-depth oscillations. Cues were consistent in one interval and in conflict in the other. We parametrically varied the difference between cues in three ways to obtain thresholds on conflict discrimination. Cue conflict was introduced by varying either the amplitude of CS relative to CD, by varying CS oscillation frequency, or by varying CS phase (Fig. 3a). Participants were asked to determine the interval in which the object physically changed in size.

Psychometric functions were fit to the data for each conflict type for 9 participants to obtain 75% correct thresholds on conflict discrimination (Fig. 3b). To compare between thresholds for different conflict types, we determined the physical surface size change required to produce threshold CS values, under the assumption that the motion-in-depth was accurately defined by CD. We simulated 1000 threshold-level conflicts for each participant (see “Methods” for details). For each simulated motion we calculated the ratio of CD to CS physical surface sizes and took the log_2_ absolute maximum as a measure of the conflict at threshold. To compare between participants and conflict types we took the mean of the simulated maximum physical size ratios for each participant. Figure 3c,d show how these equivalent physical size ratios vary across conflict types. Comparison between conflict types shows that phase conflicts were significantly easier to discern than either amplitude or frequency conflicts, with amplitude conflicts slightly easier to detect than frequency conflicts: F_2,8_ = 34.29, *p* < 0.0005, ${\eta }_{partial}^{2}=0.809$ on a Repeated Measures ANOVA, with Holm-Bonferroni corrected, related samples t-tests showing significant differences between phase and frequency (*t*_*8*_ = 18.0733, p < 0.00005, d = 4.86), phase and amplitude (*t*_*8*_ = 4.3169, p 0.0026, d = 1.68), and amplitude and frequency conflict thresholds (*t*_*8*_ = 2.8022, *p* = 0.0231, d = 1.31).

To understand these differences in conflict discrimination, we first adapted our joint distribution model to the task used in Experiment 2. Rather than combining CS and CD, we modelled conflict discrimination performance with three measurements. First, we calculated the difference in estimated distance moved between CS and CD cues at each sampled point in depth. Taking either the maximum inter-cue difference or the standard deviation of cue differences allowed the model to successfully determine the conflicting cue interval. Performance far exceeded human participants, however, and did not show equivalent differences in the effectiveness of conflict types (Fig. 3c). As a second measure, we estimated both the viewing distance and distance moved for CS and CD signals, allowing us to recover the surface sizes consistent with each cue. Conflict was measured as the difference in estimated sizes between cues. This model could not successfully discriminate the interval containing the cue conflict stimulus. Finally, we also calculated the viewing distance and distance moved under conditions of cue integration and used these to recover estimates of surface size. Using this measure, the conflict interval was selected as the interval where the standard deviation of estimated surface sizes was larger. As with the comparison of surface size estimates between cues, this approach did not support successful conflict discrimination (see “Methods” for details).

These results suggest that the visual system’s decision to integrate CS and CD cues cannot be easily attributed to differences in individual cue estimates. Such a finding is consistent with existing results showing that human adults do not retain individual, within modality, cue estimates^35^, and with our finding that thresholds for different conflict types would require different physical changes in surface size. How, then, are such conflicts detected? Since both CS and CD depend upon the same scene parameters, one possible cue to conflict may be the statistical relationship between these signals.

To examine this possibility, we measured the probability of co-occurrence of pairs of CS and CD signals, p(CS ∩ CD), using the same simulated linear motions as in our speed-in-depth discrimination model. Similar assumed or experimentally defined relationships between cues, typically referred to as ‘coupling priors’, have previously been used to account for phenomena such as multisensory temporal perception^36,37^, and the perception of material properties^38,39^. Unlike these works, our co-occurrence distribution is derived directly from simulated CS and CD signals and does not make any inferences about the likelihood of real-world motions.

**Figure 4:**
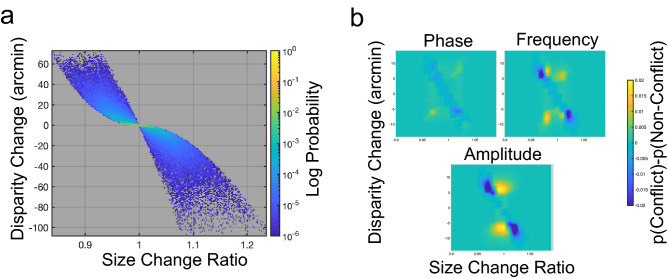
Cue Co-occurrence statistics for changing size and changing disparity (**a**). To model cue conflict discrimination, we considered the probability of co-occurrence of CD and CS cues, the distribution for which is shown here. (**b**) Comparison of cue co-occurrence for consistent cue and threshold conflict stimuli. Blue areas show combinations of CD and CS signals that are less likely to occur in threshold conflicts than in consistent cue stimuli. Yellow areas show combinations of cues that are more likely to occur under such conditions. For threshold-level conflicts, there was an increase in otherwise unlikely combinations of CD and CS, and a reduction in typically more likely combinations.

The co-occurrence distribution shows a clear relationship between CS and CD signals, with some combinations of these signals either highly unlikely or entirely absent (Fig. 4a). Larger CD and CS values are also associated with a larger range of values for the other cue. This distribution is, however, conditioned on scenes where objects do not physically change in size. As such, the relationship between cues is substantially different for the cue conflict stimuli in our experiment. To examine the effects of cue conflict, we compared the probability of CS and CD co-occurrence for the triangular depth oscillations found in both consistent cue and threshold cue conflict stimuli. This comparison showed a marked reduction in expected areas of co-occurrence for cue conflict stimuli and a substantial increase for unlikely CS and CD combinations (Fig. 4b).

Given the observed relationship between CD and CS signals, and the consistent pattern of change in this relationship evident with the introduction of each form of cue conflict, we examined whether such co-occurrence statistics, and deviation from those statistics, could explain performance in the conflict discrimination task. Our modelling work here is informed by the idea that neurons tuned to unlikely stimulus combinations could act as conflict sensitive ‘What Not?’ detectors^40–42^, where the responses of such neurons inhibit integrative processes. As before, we sampled size and disparity values every 5 frames along the motion trajectory, with signal uncertainties *n*_*disparity*_ and *n*_*size*_ as free parameters. Rather than being used to estimate speed-in-depth, these measurements were used to weight the distribution p(CS ∩ CD). The sum of this weighted distribution was taken as the probability that these signals co-occur. For each sampled CS and CD signal, this co-occurrence probability was compared to a threshold value *T*_*inhibitory*_, with subthreshold values acting as inhibitory signals and suprathreshold values acting as excitatory signals. Thus, each sampled point contributed either a single excitatory or inhibitory signal, used to determine whether cues in that stimulus were in conflict.

Cue conflict decisions were reached by finding the proportion of inhibitory signals over the total stimulus motion and comparing to a threshold value *T*_*conflict*_. Stimuli exceeding this threshold were judged as conflict stimuli, with task performance based on selecting the interval where the stimulus was in conflict. If either both or neither interval was judged as in conflict, the model made an unbiased guess. The model therefore made a discrete, conflict/no conflict judgement for each stimulus, rather than attempt to select an interval containing a greater degree of cue conflict. This cue co-occurrence model was fit to the psychophysical data using *T*_*inhibitory*_ and *T*_*conflict*_ as free parameters, in addition to the uncertainty parameters *n*_*disparity*_ and *n*_*size*_. Our fitted model matched observed threshold patterns for each conflict type (r^2^ = 0.78, *p* < 0.0005, see Fig. 3d and yellow diamond symbols on Fig. 3c). Best-fitting values for *n*_*disparity*_ and *n*_*size*_ closely matched those found in Experiment 1, averaging 0.36 arcmin and 2.50 arcmin across participants, with average *T*_*inhibitory*_ and *T*_*conflict*_ values of 1.5 × 10^–5^ and 0.15, respectively.

## Discussion

The results reported here show that the combination of CS and CD cues for motion-in-depth perception depends on the visual system understanding how these signals map onto real-world motions. This understanding includes expected CS and CD combinations, with such information being used to support cue integration processes. While other work has examined how changes in viewing geometry alter both individual cue reliabilities and their integration^34,43–45^ ours is the first to show that these changes in reliability can be directly connected to the statistical relationships linking scene and image parameters. Similarly, while existing research has sought to account for the conditions under which cue integration does and does not occur, this has largely been driven by the direct measurement of cue conflict^25^ and by the assumed mapping of estimates from one cue onto another via a ‘coupling prior’^36–39^. Here, we have shown that the statistics of co-occurrence of CS and CD cues can be used to determine integration independently of any further inference of real-world motions.

### Image statistics, scene statistics and non-standard cue integration effects

A critical main finding of our work is that non-standard cue integration effects, such as the changes in implied cue uncertainty shown in Fig. 2c, can be accounted for by an understanding of the statistical relationships between motion-in-depth cues and real-world motions. In comparison, existing models of cue integration have tended to define signal uncertainty on scales measuring the scene property under investigation^30,35^. Thus, for example, size uncertainty has been defined on a scale of millimeters^30,39^. This is a direct consequence of the standard perturbation analysis approach, where measures of uncertainty are empirically derived from the standard deviations of psychometric functions.

Our work suggests that definitions of uncertainty derived from the imaging process may be more appropriate. Here, we have defined uncertainty at both the level of image measurement reliability and the uncertainty of the mapping between scene and image properties. Similar prior suggestions have been made^34^ although, unlike our work, these did not directly model the uncertainty arising directly from the imaging process or define uncertainty at the level of image measurements. Consideration of the imaging process may be particularly useful where there is a high degree of uncertainty in the mapping between scene and image properties, as is evident in our motion-in-depth tasks. Our results show that such consideration of imaging-derived uncertainty can still occur within the general framework of independent signals used in standard approaches to cue integration.

One potentially limiting factor for this approach is the requirement to learn the distributions mapping image signals to real-world motions. Although this is a general problem for Bayesian formulated models of perception^46,47^, its resolution may be comparatively straight-forward for motion-in-depth. Given that changes in both image size and disparity occur not just when objects move independently, but also when the observer moves in the environment, these cues will correlate with both proprioceptive and vestibular cues to self-motion, in addition to real-world object motion. A correlation of this kind could be used to map CS and CD responses onto sensory signals that scale more directly with the parameters of the distal scene. Notably, combined visual-vestibular responses have been found in area MSTd of the macaque^48^, with this area implicated in the processing of three-dimensional motion, alongside area MT^49^. Indeed, MT has recently been shown to respond to motion-in-depth trajectories in a manner consistent with the encoding of direction in the real-world^50^.

A further factor not addressed in our current work is the effect of scene statistics on CS and CD distributions. Here, our simulations assumed flat prior probabilities across object size, viewing distance and movement distance, and considered only motions along the line of sight. Yet we know that motion is far more varied in natural scenes, that factors such as occlusion and perspective projection make near distances more likely than far distances^51,52^ and can likely assume that object sizes are not actually uniformly distributed. Such changes to scene statistics could increase the probability of larger CS and CD responses. This possibility should be examined in future by measuring distributions of depth and motion, and their relationship with CS and CD values in natural scenes. Similarly, disparity difference and interocular velocity difference measures of motion-in-depth show different tuning properties^17^, which could impact on cue integration. Understanding any such effects may depend on understanding how scene statistics relate to these tuning properties.

### Benefits of cue co-occurrence statistics for determining integration

In addition to showing how scene statistics affect cue integration, we have also shown how an understanding of the probability of CS and CD co-occurrence can support decisions on when to integrate these signals. This approach bears some similarity to existing causal inference models^53^ and ‘coupling priors’^36,37,39^, but, as with our cue integration model, is distinct in depending primarily on probabilities derived from the imaging process. We use these co-occurrence statistics as weights on inhibitory ‘What Not?’ detectors^40–42^, which act to gate integration processes. Notably, the alternative strategy of measuring cue conflict in terms of scene parameter estimates fails to account for our psychophysical results, despite the success of such models of ‘robust integration’ in other domains^25^. This may initially seem like an odd strategy: why would the visual system opt to infer cue conflict from unlikely signal combinations, rather than measure it directly? One possibility is that co-occurrence statistics offer a simpler means of assessing conflict that does not depend upon the potentially unnecessary further processing of motion-in-depth information.

### Perceptual effects of large cue conflicts

A peculiar finding from our work is that while cue weighting tends towards CS, this is not reflected in the perceptual consequences of large conflicts. Under such large conflict conditions, objects are instead perceived as changing in physical size. Given the relative preference for CS signals when cues are integrated, one might expect large conflicts to result in the perception of multiple object motions, consistent with a less reliable CD cue. That this was not the case further supports the idea that decisions on whether to integrate multiple cues are distinct from the processes underpinning integration. These results are also in agreement with earlier findings showing that perception does not always follow the more reliable cue when conflicts are large^25,26^. Instead, the perceptual consequences of large conflicts seem better accounted for by a system that ‘explains away’ conflicting signals^54^. Such an explanation requires the visual system to assess the relative reliability of the prior assumptions of object size constancy and multiple object motions, upon which CS and CD estimates depend. Large cue conflicts may then be ‘explained away’ by abandoning the less reliable assumption, namely that the object has not physically changed in size. While some earlier research^55^ has examined the effects of manipulating prior probabilities, significant further work is required to understand the role they play in resolving sensory conflict.

## Conclusions

To make full use of the information available in changing size and changing disparity cues, the visual system must consider the way that these signals map onto real-world motions. Our results show that consideration of these statistical properties plays a critical role in both the decision to integrate cues, and in how they are used to infer motion-in-depth. Together, they suggest that the decision to integrate motion-in-depth cues precedes the translation of these signals into estimates of real-world motion. These results provide new insight into the visual system’s understanding of the uncertainty arising from the imaging process and provide a demonstration of how such uncertainty impacts sensory cue integration and the perception of motion-in-depth.

## Methods

### Participants

Participants were all staff or students at the University of Stirling. Experiment 1 was completed by 6 participants, including authors RG and LM, while Experiment 2 was completed by 9 participants, including all 3 authors. The authors were the only participants to complete both experiments. All participants had normal or corrected-to-normal vision. All non-author participants were naïve as to the aims of the experiments and received compensation at standard university rates or completed the experiment in partial fulfilment of course requirements. Participants gave written, informed consent, with all experimental procedures approved by the University of Stirling general ethics panel in accordance with British Psychological Society guidelines and the Declaration of Helsinki.

### Stimuli & apparatus

Experiments were programmed in Matlab (© Mathworks Inc. Natick, MA, USA), in conjunction with the Psychophysics Toolbox extensions^56–58^. Stimulus presentation was controlled using a MacPro computer, with stimuli displayed on a 49 × 31 cm Apple HD Cinema display (© Apple Inc., Cupertino, CA, USA) with a resolution of 1920 × 1200 pixels and a refresh rate of 60 Hz. Each pixel subtended an angle of 1.1arcmin at the 76.4 cm viewing distance. The display was calibrated using a SpyderPro2 calibration device (© DataColor, Dietlikon, Switzerland) to ensure a linear greyscale for luminance. Luminance range was 0.18 cdm^-2^ to 45.7 cdm^-2^. Presentation of binocular disparities was enabled through the use of a 4-mirror modified Wheatstone stereoscope, calibrated to ensure vergence and accommodation were consistent with the viewing distance. Stimuli were displayed in a darkened lab to minimise visibility of other objects. Head movements were controlled using a Headspot chinrest (© UHCO, Houston, TX, USA).

The basic stimulus for all experiments was a flat, fronto-parallel square, textured with a 12 × 12 random luminance checkboard pattern. This pattern was scaled depending on object size and distance to ensure both consistency of the CS cue and that number of checks could not be used as a proxy cue to object size. All objects moved through a triangular wave in depth, directly towards and away from the observer along the line of sight. The amplitude of the triangular wave was 80 mm. Baseline speed-in-depth was 213 mm/s, with stimuli presented for 1500 ms. This translated to a single pass through the complete triangular motion. Simulated surface size, given by the length of one side of the square surface, was 10, 40 or 60 mm in Experiment 1, and 40 mm in Experiment 2. At the plane of fixation, these corresponded to angular sizes of 45, 180 and 270 arcmin.

To complicate participants’ ability to use ancillary cues such as presentation duration or total change in image size/disparity when judging motion-in-depth, baseline motion amplitudes, surface sizes and presentation durations were varied at random by ± 20 mm, ± 10 mm and ± 67 ms, respectively. Surface size was randomly varied in Experiment 2 only. Each stimulus was preceded by the 500 ms presentation of a fixation cross. Moving stimuli were flanked, at an angular distance of 6.12° above and below the centre of the screen, by a pair of stationary square checkerboard patterns, situated at the fixation plane. These were structurally equivalent to the moving surfaces, except that they were at a constant simulated size of 20 mm, giving them an angular size of 90 arcmin.

For all stimuli, CS and CD information was added by back projecting the simulated object to the plane of the screen under perspective projection. To allow for the addition of conflict between CS and CD signals, disparity information was calculated based on the centre of the object only. Although this had the potential to introduce errors in simulated disparities at object edges, and necessitated the absence of vertical disparities, in practice the comparatively small object sizes and limited range of, foveally presented, motion ensured that these issues were not relevant in our experiments.

Measurements of CD and CS were taken as the difference in disparity and the ratio of sizes, respectively. CD measures were thus given by the following equation:

1 $$CD = 2{\text{tan}}^{{ - 1}} \left( {\frac{{i/2}}{v}} \right) - 2{\text{tan}}^{{ - 1}} \left( {\frac{{i/2}}{{v - m}}} \right)$$

for the interocular separation *i,* the viewing distance *v* and the distance moved *m,* for a point moving forwards and backwards along the line of sight. Note that the order of the subtraction maintains the signing convention where negative disparity values indicate crossed (i.e., closer) disparities.

CS ratio values were given by the following equation:

2 $$CS = \frac{{2\tan ^{{ - 1}} \left( {\frac{{s/2}}{{\nu - m}}} \right)}}{{2\tan ^{{ - 1}} \left( {\frac{{s/2}}{\nu }} \right)}}$$

where *s* is the surface size. Ratios greater than 1 indicate in increase in image size. As with CD estimates, this equation holds for motion along the line of sight only. Note that, unlike the equation for CD, CS values are dependent upon the surface size, although, due to the ratio of arctangents, this effect is reduced for smaller surface sizes.

In both experiments, the consistency of CS and CD signals was manipulated to put these cues into conflict. Cue conflict was added in three ways, through manipulation of the amplitude of the triangular waveform of one or both cues, the frequency of the waveform, or the relative phase (Fig. 3a). Experiment 1 used only amplitude conflict, while all three forms were used in the conflict discrimination task in Experiment 2. In Experiment 1, conflict was set at values of ± 0.32 log_2_ units of the standard amplitude. The direction of conflict was counterbalanced across CS and CD cues. Conflict manipulations in Experiment 2 were applied to the CS cue only, such that the amplitude, frequency or phase of the CS cue was varied relative to the standard value at which the CD cue was set.

### Experiment 1: speed-in-depth discrimination

#### Design and procedure

Speed-in-depth discrimination was measured using the perturbation analysis paradigm^30,31^. Participants were presented with pairs of stimuli, in a two-interval forced choice (2IFC) design, where one, randomly selected, interval contained a stimulus with conflicting CS and CD cues. As described above, conflict was introduced through the manipulation of the amplitude of motion-in-depth for each cue. The other interval contained a consistent cue stimulus, the speed of which was varied randomly, on a trial-by-trial basis, across 7 equally spaced speed ratios of between ± 1.2 log_2_ units of the standard amplitude. Participants’ task was to report the interval containing the faster motion. Each participant completed a minimum of 30 repeated trials of each stimulus level. Responses were used to calculate the proportion of trials on which the consistent cue stimulus was judged to be faster than the conflict cue stimulus, with data fitted to a cumulative Gaussian function, described by the mean μ and standard deviation σ. Mean values corresponded to the point of subjective equality (PSE), while the standard deviation gave the just noticeable difference (JND), equivalent to the 84% point on the function. We compared these parameters to those obtained from a model of cue integration that considers the statistical relationship between real-world motions and resulting CD and CS signals.

#### Implied cue uncertainty

The perturbation analysis approach applied in Experiment 1 is commonly used to test standard maximum likelihood models of linear cue integration^30,31^. Typically, this approach predicts combined cue PSEs and JNDs from performance in single cue conditions, under the assumption that cue integration seeks to minimise sensory uncertainty. Here, we were concerned that such single cue conditions contained significant cue conflicts that could disrupt motion-in-depth perception. As an alternative, we used the standard maximum likelihood approach to derive equations for the *implied uncertainty* of individual cues, given PSE locations and combined cue JNDs. This measure thus offers an alternative test of the maximum likelihood model, where deviations from expected uncertainty relationships may be evident in implied uncertainty values.

Implied single cue uncertainty values were calculated using established relationships between single cue weights (derived from PSE locations) and JNDs, under the assumption of maximum likelihood consistent integration. Weights *w* were derived from PSEs using the following:

3 $${w}_{CD}=\frac{\left(PSE-s\right)}{\left(d-s\right)}, { w}_{CS}=1-{w}_{CD}$$

where *s* and *d* are, respectively, the speeds in depth of the CS and CD cues in the conflict stimulus.

Using the following relationship^30^ between signal weights and single cue uncertainties given by JNDs/function slopes σ,

4 $$\frac{{w}_{CD}}{{w}_{CS}}=\frac{{\sigma }_{CS}^{2}}{{\sigma }_{CD}^{2}}$$

and its rearranged form

5 $${\sigma }_{CS}^{2}={\sigma }_{CD}^{2}\frac{{w}_{CD}}{{w}_{CS}}$$

We calculated the implied uncertainty for each cue using the relationship between single and combined cue uncertainties^26^,

6 $${\sigma }_{CD\_CS}^{2}=\frac{{\sigma }_{CD}^{2}{\sigma }_{CS}^{2}}{{\sigma }_{CD}^{2}+{\sigma }_{CS}^{2}}$$

Substituting ${\sigma }_{CD}^{2}\frac{{w}_{CD}}{{w}_{CS}}$ for ${\sigma }_{CS}^{2}$, this gives the following equation,

7 $${\sigma }_{CD\_CS}^{2}=\frac{{\sigma }_{CD}^{4}\frac{{w}_{CD}}{{w}_{CS}}}{{\sigma }_{CD}^{2}+{\sigma }_{CD}^{2}\frac{{w}_{CD}}{{w}_{CS}}}$$

which may be rearranged in terms of ${\sigma }_{CD}^{2}$, as follows

$${\sigma }_{CD\_CS}^{2}\left[{\sigma }_{CD}^{2}+{\sigma }_{CD}^{2}\frac{{w}_{CD}}{{w}_{CS}}\right]={\sigma }_{CD}^{4}\frac{{w}_{CD}}{{w}_{CS}}$$

$${\sigma }_{CD}^{2}\left[{\sigma }_{CD\_CS}^{2}+{\sigma }_{CD\_CS}^{2}\frac{{w}_{CD}}{{w}_{CS}}\right]={\sigma }_{CD}^{4}\frac{{w}_{CD}}{{w}_{CS}}$$

to give final equations for ${\sigma }_{CS}^{2}$ and ${\sigma }_{CD}^{2}$

8 $${\sigma }_{CD}^{2}=\frac{{w}_{CS}}{{w}_{CD}}{\sigma }_{CD\_CS}^{2}+{\sigma }_{CD\_CS}^{2}, {\sigma }_{CS}^{2}={\sigma }_{CD}^{2}\frac{{w}_{CD}}{{w}_{CS}}$$

These equations depend solely on measured combined cue JNDs and on the ratio of cue weights, as derived from measured PSE locations. We compared these implied uncertainties to those expected when CD uncertainties remained constant. These uncertainties were calculated using measured PSE locations to define the weight ratio $\frac{{w}_{CD}}{{w}_{CS}}$, alongside the implied CD uncertainty for the smallest surface size. This allowed the calculation of implied CS uncertainty using Eq. (8). Note that the choice of implied CD uncertainty here is arbitrary. This comparison simply illustrates the expected pattern of results given that our manipulation of surface size should only have affected CS uncertainty.

### Experiment 2: cue conflict discrimination

#### Design and procedure

Conflict discrimination was measured in Experiment 2 by varying the extent of the amplitude, frequency or phase conflict between CS and CD cues. As in Experiment 1, participants were presented with a 2IFC task, although here they were asked to select the interval where the stimulus appeared to physically change in size. This task was selected following initial subjective evaluation of conflict stimuli, where participants consistently reported the appearance of such size changes. In each case, one randomly selected interval contained a stimulus where CS and CD cues were consistent with one another, while the other contained a stimulus where these cues were in conflict. Cue conflict was parametrically varied across 7 equally space levels for three conflict types: amplitude, frequency and phase. Amplitude ratios varied between ± 1, frequency ratios ranged from -2 to 0 log_2_ units, and relative phase varied between 0 and π. Participants completed a minimum of 30 repeated trials of each stimulus level. Responses for Experiment 2 were used to calculate proportion correct scores, fitting scaled cumulative Gaussian functions for each conflict type. The 75% correct point on the function was taken as the threshold measure of performance.

#### Equivalent physical size ratios

In order to compare discrimination performance for each conflict type, where each is measured on a different scale, we reinterpreted measured threshold values in terms of the physical change to surface size that would have resulted in an equivalent cue conflict. Equivalent physical sizes were calculated by assuming that motion-in-depth was accurately signalled by the CD cue, allowing the size of the surface to be measured for each stimulus frame using the image size and measured disparity. Where the viewing distance *v* and distance moved *m*_*CD*_ for the CD cue are known, the surface size can be calculated as ${s}_{CD}=2\left(v-{m}_{CD}\right)\mathrm{tan\theta }$, where θ is half the angular size of the surface, as given by the CS cue via the equation $\theta ={\mathrm{tan}}^{-1}\left[\left({s}_{CS}/2\right)/\left(v-{m}_{CS}\right)\right]$. Note that we do not assume that these measures are available to observers; this is simply a means of interpreting the measurable conflict for any stimulus.

We generated these measures of equivalent surface size for 1000 threshold-level conflicts for each participant, with the addition of randomised adjustments to surface size, amplitude-in-depth, and speed-in-depth as was the case in Experiment 2. Using these measures, we found the equivalent physical size ratio of the conflicting surface size *s*_*CD*_, to actual simulated surface size s_CS_, on a log_2_ scale (i.e., ${log}_{2}\left[{s}_{CD}/{s}_{CS}\right]$) for each frame of each simulated threshold-level conflict. Equivalent physical size ratios were summarised for each simulated motion as the maximum absolute log_2_ ratio across frames, taking the mean of this measure across the 1000 simulations for each participant.

### Cue integration models

The novel cue integration models presented in our paper address two distinct problems of motion-in-depth perception, the discrimination of speed-in-depth, and the detection of cue conflict. In each case, our models depend upon probability distributions of CS and CD signals produced from Monte Carlo simulations of linear motions in depth. Below, we describe these simulations in detail, followed by the different models that make use of the statistical information they provide.

#### Simulations of motion-in-depth

Monte Carlo simulations were conducted for 1 million linear motions of a fronto-parallel surface moving directly towards or away from an observer. To understand how object motions map onto CS and CD cues, we performed simulations using a range of viewing distances, surface sizes and amounts of motion. Viewing distances were chosen at random from a range of 0.5 m to 10 m, with surface sizes ranging from 2 to 1000 mm. Distance moved varied at random between a range of ± 100 mm. Each motion was defined as start and end positions only, with all motions starting at the point of fixation. As such, our simulations do not provide information for the calculation of motion energy, or other more direct measures of spatiotemporal change.

Simulations were used to define the joint posterior distributions p(v,m|CD) and p(v,m|CS) for the viewing distance *v* and distance moved *m*, given the signal from the CD or CS cue. CS values closer to one were consistent with a large range of viewing distances, but a more limited range of distances moved. Joint distributions for CD showed that smaller CD values were consistent with both a wide range of viewing distances and of distances moved. For both CS and CD cues, larger values signalled smaller viewing distances, but were consistent with a wide range of real-world motions. Examples of these distributions are shown in Fig. 1b.

Joint posterior distributions were used to estimate the viewing distance *v* and distance moved *m* for 10,000 new linear motion simulations. Combined cue posterior distributions were taken as the normalised product of individual cue posteriors. Combined cue estimates for viewing distance and distance moved were taken as the marginal means for each parameter.

#### Speed-in-depth discrimination

We modelled the discrimination of speed-in-depth by introducing uncertainty to initial CD and CS measurements, then applying our joint distribution approach. Disparity and image size values were sampled along each cue’s triangular oscillation in depth, with uncertainty added by defining these cues as unbiased Gaussian distributions with standard deviations *n*_*disparity*_ and *n*_*size*_, respectively. CD measures were given by the disparity difference between sampled frames, with *n*_*disparity*_ giving the standard deviation of the uncertainty on each frame, resulting in a CD measure with a variance of $2{n}_{disparity}^{2}$. CS was measured as the ratio of image sizes between sampled frames, resulting in a CS measure with a variance defined by the ratio distribution between points^32^.

The resulting CD and CS distributions were used to weight individual cue distributions to produce posterior distributions for *m* and *v* between each sampled point. Posterior distributions were defined as the weighted sum of distributions, normalised by the sum of these functions. This is shown for the CD cue in Eq. (1) below.

9 $${p}_{weighted}\left(m,v|CD\right)=\frac{B}{\sum_{\mathrm{min}(m,v)}^{\mathrm{max}(m,v)}B},\mathrm{ where }B=\sum_{x=1}^{x=n}{L}_{CD}\left(x\right)p\left(m,v|CD\right)$$

where *L*_*CD*_ defines the weight for each potential CD value *x* (from 1st to nth index on these values). *L*_*CS*_ (see Fig. 1b) defines the equivalent weightings for each CS value.

Combined cue estimates were generated by multiplying together the weighted posterior distributions for each cue and renormalising as in Eq. (2).

10 $$p\left(m,v|Combined\right)=\frac{{p}_{weighted}\left(m,v|CD\right){p}_{weighted}\left(m,v|CS\right)}{\sum {p}_{weighted}\left(m,v|CD\right)+{p}_{weighted}\left(m,v|CS\right)}$$

Finally, speed-in-depth estimates were found by taking the marginal mean *M* of combined cue distributions at each sampled point in depth with respect to distance moved, summing the estimated absolute motions, and dividing by the presentation duration *t,* i.e., $\sum \left|M\right|/t$. This model was fit to human task performance, using joint likelihood distributions, with *n*_*disparity*_ and *n*_*size*_ as free parameters. Fitted models minimised the mean square error between model and human performance for all tested speed ratios and object sizes.

#### Cue conflict discrimination

Our modelling of cue conflict discrimination began by adapting the joint distribution model detailed above. This adapted model measured three key aspects of stimulus conflict. Our first measure was the difference in estimated distance moved between CS and CD cues at each sampled stimulus point. The conflict interval was selected by finding the interval containing the larger mean or maximum difference in motion estimates between cues. Our second measure used the joint distribution model to estimate both viewing distance and distance moved for CS and CD signals, allowing for the calculation of estimated surface size at each sampled stimulus point. Mean and maximum surface size differences between cues were taken as measures of cue conflict, with the model selecting the interval containing the larger difference. Finally, combined cue estimates of viewing distance and distance moved were used to calculate surface size at each sampled point, with conflict measured as the standard deviation of estimated surface sizes. The interval containing the larger standard deviation was selected as the conflict stimulus.

In addition to these models, we also modelled conflict discrimination by considering the probability of co-occurrence of pairs of CS and CD signals. We measured the distribution of such co-occurrences p(CS ∩ CD), using the same simulated linear motions that defined our speed-in-depth joint distributions. To model conflict discrimination, we again sampled size and disparity values every 5 frames along the motion trajectory, with signal uncertainties *n*_*disparity*_ and *n*_*size*_ as free parameters. Signal uncertainties were used to weight the distribution p(CS ∩ CD), with the probability of co-occurrence for each sampled point taken as the sum of the co-occurrence distribution multiplied by the distribution of uncertain CS and CD signals.

For each sampled stimulus point, the co-occurrence probability was compared to a threshold value *T*_*inhibitory*_. Co-occurrence values below this threshold were taken as inhibitory signals, with suprathreshold values acting as excitatory signals, providing a binary inhibitory/excitatory response at each point along the motion trajectory.

Cue conflict decisions were reached by comparing the proportion of inhibitory signals over the total stimulus motion to a threshold value *T*_*conflict*_. Stimuli that exceeded this threshold were judged as conflict stimuli, with task performance based on selecting the interval where the stimulus was in conflict. If either both or neither interval was judged as in conflict, the decision was reached via an unbiased guess. This means that our model made a discrete, conflict/no conflict judgement for each stimulus, rather than attempt to select an interval containing a greater degree of cue conflict. Free parameters for this model were the uncertainty parameters *n*_*disparity*_ and *n*_*size*_*, and the threshold parameters T*_*inhibitory*_ and *T*_*conflict*_.

## Data Availability

The datasets used in this study, along with associated analysis code, are available from the corresponding author on reasonable request.
